# Prevalence and Risk Factors of Gallbladder Stones and Polyps in Liaoning, China

**DOI:** 10.3389/fmed.2022.865458

**Published:** 2022-04-25

**Authors:** Xinhe Zhang, Lin Guan, Haoyu Tian, Yiling Li

**Affiliations:** ^1^Gastroenterology Department, The First Affiliated Hospital of China Medical University, Shenyang, China; ^2^The Third Clinical Department, China Medical University, Shenyang, China

**Keywords:** gallbladder stones, gallbladder polyps, prevalence, risk factors, fatty liver, metabolic syndrome

## Abstract

**Objective:**

To investigate the incidence and risk factors of gallbladder stones and polyps in individuals undergoing physical examinations in Liaoning province, China.

**Methods:**

This is a retrospective study of adults who underwent routine health examinations at Xikang Medical Center in Liaoning Province (Shenyang, Dandong, and Dalian) from 01/2016 to 12/2020. The routine health examination included anthropometry, blood tests, and liver ultrasound. Based on liver ultrasound results, patients were grouped into those with gallbladder stones, those with gallbladder polyps, those with both stones and polyps, and those with neither.

**Results:**

Of the 284,129 included subjects, 6,537 (2.30%) were diagnosed with gallbladder stones, and 18,873 (6.64%) were diagnosed with gallbladder polyps. The overall prevalence in Liaoning province increased each year, peaking in 2020. The prevalence of gallbladder stones was higher among females than males (2.39% vs. 2.23%, respectively), while the prevalence of gallbladder polyps was higher among males. The gallbladder polyp group had higher BMI, FBG, SBP, DBP, TG, TC, LDL-C, HDL-C, AST, ALP, GGT, BUN, Scr, SUA. Except for HDL-C, all factors were also higher in the gallbladder stone group. Patients with fatty liver had a higher prevalence of gallbladder stones and polyps than participants without fatty liver.

**Conclusion:**

The prevalence of gallbladder stones and polyps in Liaoning varies by sex, economic status of the city of residence, BMI, and metabolic indicators.

## Introduction

Gallbladder stones are the most common form of gallbladder disease, with a high prevalence among middle-aged women. The disorder is caused by particular contents in the bile. While the proportion of cholesterol is higher, the proportion of bile acid content is relatively lower, resulting in the long-term crystallization of cholesterol into stones (1). Most patients with gallbladder stones have asymptomatic gallstones with no clinical symptoms (2), but some experience symptoms such as discomfort in the right upper abdomen, dyspepsia, and biliary colic. Patients do not usually seek medical care for atypical symptoms. The most common clinical manifestation of gallbladder stones is biliary colic (3). This may occur after patients eat greasy food or change position, causing persistent pain in the right upper abdomen with paroxysmal exacerbation and dissipation to the right shoulder. Biliary colic occurs because stones are embedded in the neck of the gallbladder, preventing bile from being discharged and causing strong contractions. If gallbladder stone incarceration is not diagnosed or treated in time, the patient may develop acute cholecystitis with infection. This can lead to pancreatitis if gallbladder stones fall into the common bile duct. Another common gallbladder disease is gallbladder polyps, characterized as a local protrusion of the inner wall of the gallbladder into the gallbladder cavity. Its main etiology and clinical manifestation are similar to that of gallbladder stones. As a result of the increased cholesterol content in the bile (4), macrophages aggregate and phagocytose in this region, gradually forming protrusions that protrude out of the mucosal surface (5).

In recent years, there has been an increasing prevalence of gallbladder stones and polyps. The current prevalence of gallbladder stones has risen to >10%. This study retrospectively analyses physical examination data from 3 major cities in Liaoning Province, Shenyang, Dalian, and Dandong, to assess the prevalence of gallbladder stones and polyps in this region, and further explore their related risk factors.

## Materials and Methods

### Study Subjects

This is a retrospective study of adults who underwent routine health examinations at Xikang Medical Center in Liaoning Province between January 2016 and December 2020. Based on the population distribution of Liaoning Province, three cities with the largest populations, Shenyang, Dalian, and Dandong, were selected for study. Individuals who were >18 years of age, were living in Shenyang, Dalian, or Dandong for at least 5 years, had participated in the annual physical examination, and had no missing data were included in the study. For individuals with more than one examination during the study period, only the first examination was included. Overall, 2,84,129 individuals were included in the study and divided into the gallbladder stone only group (sGS), the gallbladder polyps only group (sGP), the gallbladder stone and polyps group (GSP), and the no gallbladder stones or polyps group (nGSP). The study was conducted in accordance with the Declaration of Helsinki, and the protocol was approved by the Ethics Committee of the First Hospital of China Medical University ([2020]2020-257). The ethics committee waived the requirement for informed consent because of the retrospective nature of the study.

### Physical Examinations

All physical examinations and blood tests included in this study were part of the routine examination. Blood pressure measurements, including systolic blood pressure (SBP) and diastolic blood pressure (DBP), were taken twice using an electronic sphygmomanometer (HEM-7200, OMRON Healthcare, Kyoto, Japan) after the participants had been sitting in a calm state for at least 5 min. Height and weight were measured while fasting in the morning, and body mass index (BMI) was measured as kg/m^2^. Anterior cubital vein blood was drawn in the fasting state (at least 8 h). Midstream specimen of morning urine was also taken. Routine blood panel, liver function, kidney function, serum uric acid (SUA), fasting blood glucose (FBG), blood lipids, and routine urine analysis were assessed using a 7,600 autoanalyzer (Hitachi, Tokyo, Japan).

### Diagnostic Standards

Ultrasound was part of the routine examination and was performed by 2 experienced ultrasound radiologists with at least 5 years of experience using an IU 22 system (Philips, Best, Netherlands). An individual was diagnosed with gallbladder stones when the ultrasound examination showed a strong echogenic light group in the gallbladder followed by a sound shadow, which moved with a change in body position. An individual was diagnosed with gallbladder polyps when the ultrasound examination showed that papillary or mulberry-like nodules protruded into the gallbladder with strong or medium echoes and did not change with body position. An individual was diagnosed with fatty liver when the ultrasound examination showed that the liver had fatty liver changes (hyperechogenicity due to increased acoustic interface caused by the intracellular accumulation of lipid vesicles, blurring of vascular margins, increased liver size, and increased acoustic attenuation). Dyslipidemia diagnosis was determined using the “Guidelines for the Prevention and Treatment of Dyslipidemia in Chinese Adults” developed by the Chinese Cardiovascular Disease Association in 2007. Diabetes (DM) and hypertension diagnoses were made using 1999 World Health Organization standards. Obesity and overweight diagnoses were made using WHO recommendations (overweight: BMI ≥24 kg/m^2^; obesity: BMI ≥28 kg/m^2^).

### Statistical Analysis

Statistical analysis was performed using R 3.5.3 and R commander 2.5-3. Continuous variables were expressed as the mean ± standard deviation and the parameter *t*-test was used to assess the difference between groups. The counting data were expressed as percentages and compared using a Chi-square test. Multivariate logistic regression was used for the risk factors analysis. *P*-values <0.05 were considered statistically significant in all analyses.

## Results

**FIGURE 1:**
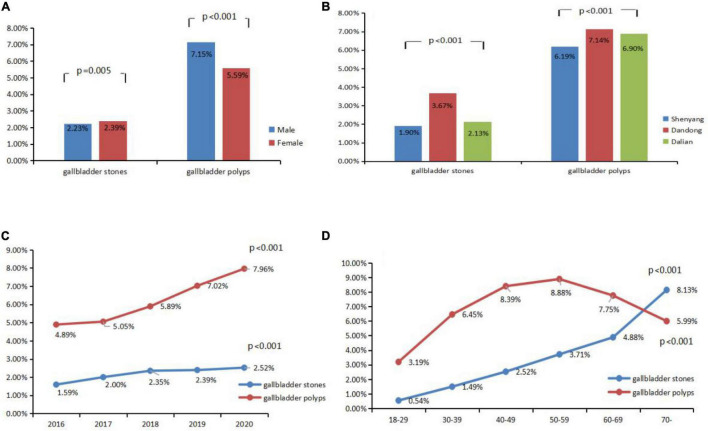
Prevalence of gallbladder stones and gallbladder polyps stratified by different subgroups **(A)** gender; **(B)** city; **(C)** year; **(D)** age.

Of the 2,84,129 included subjects, 6,537 (2.3%) were diagnosed with gallbladder stones and 18,873 (6.64%) were diagnosed with gallbladder polyps. The prevalences stratified by sex, city, time, and age are shown in Figure 1. The prevalence of gallbladder stones in Shenyang, Dandong, and Dalian in the past 5 years was 1.90, 3.67, and 2.13%, respectively, and the prevalence of gallbladder polyps was 6.19, 7.14, and 6.90%, respectively. They were significantly different across city groups (*P* < 0.001). The total annual prevalence of gallbladder stones from 2016 to 2020 was 1.59, 2.00, 2.35, 2.39, and 2.52%, respectively while the total annual prevalence of gallbladder polyps was 4.89, 5.05, 5.89, 7.02, and 7.96%, respectively. Thus, the overall prevalence in Liaoning province increased by year, peaking in 2020. The prevalence conformed to the linear and growth models using trend prediction analysis (*P* < 0.001). Gallbladder stone prevalence was higher in females than males (2.39% vs. 2.23%, respectively) (*P* < 0.05), while gallbladder polyp prevalence was higher in males than females (7.15% vs. 5.59%, respectively) (*P* < 0.001). Their prevalence was significantly different across age groups (*P* < 0.001). The prevalence of gallbladder stones increased with age, but the prevalence of gallbladder polyps peaked in patients 50–59 years of age.

Gallbladder polyp prevalence was higher in male than female patients regardless of city of residence, year, and age (except for patients >70 years) (*P* < 0.01). Gallbladder stone prevalence showed no statistical difference by year for both males and females (*P* > 0.05). The prevalence of gallbladder polyps was higher for females than males in Shenyang and Dalian but different in Dandong (*P* < 0.01). A comparison of male and female gallbladder polyp prevalence stratified by age, city, and year is shown in Table 1.

**TABLE 1:** Comparision of prevalence of gallbladder stones and gallbladder polyps between male and female stratified by different subgroups.

		Gallbladder stones			Gallbladder polyps		
		Male	Female	*P*-value	Male	Female	*P*-value
Age	18–29	158/32736(0.48%)	137/21795(0.63%)	0.023	1154/32736(3.53%)	584/21795(2.68%)	<0.001
	30–39	766/55990(1.37%)	746/45617(1.64%)	<0.001	4294/55990(7.67%)	2255/45617(4.94%)	<0.001
	40–49	742/29223(2.54%)	659/26364(2.50%)	0.767	2748/29223(9.40%)	1914/26364(7.26%)	<0.001
	50–59	853/21168(4.03%)	646/19213(3.36%)	<0.001	2198/21168(10.38%)	1390/19213(7.23%)	<0.001
	60–69	595/11793(5.05%)	564/11973(4.71%)	0.213	1004/11793(8.51%)	837/11973(6.99%)	<0.001
	70–	345/4340(7.95%)	326/3917(8.32%)	0.535	268/4340(6.18%)	227/3917(5.80%)	0.468
City	Shenyang	1197/67666(1.77%)	1080/52171(2.07%)	<0.001	4585/67666(6.78%)	2827/52171(5.42%)	<0.001
	Dandong	979/24490(4.00%)	829/24812(3.34%)	<0.001	2048/24490(8.36%)	1477/24812(5.95%)	<0.001
	Dalian	1283/63094(2.03%)	1169/51896(2.25%)	0.010	5033/63094(7.98%)	2903/51896(5.59%)	<0.001
Year	2016	201/13193(1.52%)	175/10442(1.68%)	0.352	752/13193(5.70%)	403/10442(3.86%)	<0.001
	2017	438/23020(1.90%)	438/20683(2.12%)	0.109	1283/23020(5.57%)	926/20683(4.48%)	<0.001
	2018	681/29479(2.31%)	604/25105(2.41%)	0.462	2025/29479(6.87%)	1192/25105(4.75%)	<0.001
	2019	802/34021(2.36%)	786/32394(2.43%)	0.561	2728/34021(8.02%)	1936/32394(5.98%)	<0.001
	2020	1337/55537(2.41%)	1075/40255(2.67%)	0.010	4878/55537(8.78%)	2750/40255(6.83%)	<0.001

*Data were expressed as [number of patients/number of participants (prevalence)].*

The biomarkers of each group are shown in Table 2. The gallbladder polyp group had higher BMI, FBG, SBP, DBP, TG, TC, LDL-C, HDL-C, AST, ALP, GGT, BUN, Scr, and SUA than the group with no gallbladder stones or polyps (*P* < 0.05). The same was true of the gallbladder stone group, except that there was no difference in HDL-C (P = 0.118). However, patients with both gallbladder stones and polyps had higher BMI, FBG, SBP, DBP, TG, TC, LDL-C, GGT, SUA, and Scr (*P* < 0.05).

**TABLE 2:** Comparison of related factors of gallbladder stones and gallbladder polyps.

	sGS	sGP	GSP	nGSP	*P*^1^ value	*P*^2^ value	*P*^3^ value
BMI (kg/m2)	25.07 ± 4.56	24.03 ± 3.89	25.64 ± 4.37	23.78 ± 11.22	<0.001	<0.001	<0.001
FBG (mmol/L)	5.66 ± 1.81	5.38 ± 1.33	5.80 ± 2.20	5.3 ± 1.37	<0.001	<0.001	0.015
SBP (mmHg)	129.18 ± 24.66	122.67 ± 19.94	130.39 ± 19.99	121.13 ± 20.09	<0.001	<0.001	<0.001
DBP (mmHg)	76.19 ± 15.80	72.75 ± 13.50	77.25 ± 13.50	71.5 ± 13.20	<0.001	<0.001	<0.001
TG(mmol/L)	1.57 ± 1.37	1.41 ± 1.19	1.66 ± 1.14	1.34 ± 1.24	<0.001	<0.001	0.003
TC (mmol/L)	4.7 ± 1.30	4.54 ± 1.24	4.64 ± 1.28	4.35 ± 1.46	<0.001	<0.001	0.018
LDL-C (mmol/L)	2.18 ± 1.54	2.18 ± 1.43	2.41 ± 1.40	2.03 ± 1.44	<0.001	<0.001	0.004
HDL-C (mmol/L)	0.99 ± 0.68	1.05 ± 0.66	1.00 ± 0.60	1.02 ± 0.69	0.118	0.004	0.789
ALT (U/L)	28.85 ± 28.54	26.59 ± 22.88	26.79 ± 16.70	26.68 ± 24.87	0.006	0.718	0.966
AST (U/L)	22.01 ± 19.12	20.65 ± 11.23	20.18 ± 8.01	20.13 ± 13.29	<0.001	0.019	0.965
ALP (U/L)	10.77 ± 26.52	7.44 ± 21.93	6.28 ± 21.00	6.33 ± 22.07	<0.001	0.011	0.870
GGT (U/L)	30.34 ± 57.64	25.26 ± 29.92	28.59 ± 28.23	23.17 ± 30.39	<0.001	<0.001	0.027
BUN (mmol/L)	4.64 ± 2.00	4.51 ± 1.84	4.58 ± 1.82	4.23 ± 2.67	<0.001	<0.001	0.055
Scr (μmol/L)	58.6 ± 29.03	60.1 ± 23.85	60.79 ± 23.32	56.44 ± 25.91	0.004	<0.001	0.046
SUA (μmol/L)	329.41 ± 125.54	326.69 ± 121.71	340.78 ± 134.14	311.41 ± 138.64	<0.001	<0.001	0.023

*P^1^ value, comparision between sGS group and nGSP group; P^2^ value, comparision between sGP group and nGSP group; P^3^ value, comparision between GSP group and nGSP group; BMI, body mass index; FBG, fasting blood glucose; SBP, systolic blood pressure; DBP, diastolic blood pressure; HCT, hematocrit; MCV, mean corpusular volume; TG, triglyceride; TC, serum total cholesterol; LDL-C, low-density lipoprotein cholesterol; HDL-C, high-density lipoprotein cholesterol; ALT, alanine aminotransferase; AST, aspartate aminotransferase; ALP, alkaline phosphatase; GGT, gama-glutamyl transpeptadase; BUN, blood urea nitrogen SUA, serum uric acid; Scr, serum creatinine.*

Results of the multivariable logistic analyses are shown in Table 3. In all subjects, BMI, FBG, SBP, TC, and LDL-C were independent risk factors associated with gallbladder stones (*P* < 0.05), while DBP, TC, LDL-C, and Scr were independent risk factors and for gallbladder polyps (*P* < 0.05). HDL-C level was an independent factor for both gallbladder stones and polyps with a negative association (*P* < 0.05).

**TABLE 3:** Multivariate analysis of risk factors of gallbladder stones and gallbladder polyps.

	sGS			sGP			GSP		
	Estimate	Standard Error	*P-*value	Estimate	Standard Error	*P-*value	Estimate	Standard Error	*P-*value
Age (years)	0.042	0.002	<0.001	0.018	0.001	<0.001	0.057	0.002	<0.001
BMI (kg/m2)	0.002	0.001	0.027	0.001	0.001	0.537	0.002	0.002	0.322
FBG (mmol/L)	0.094	0.016	<0.001	0.018	0.013	0.180	0.114	0.047	0.015
SBP (mmHg)	0.007	0.002	0.001	−0.002	0.002	0.254	0.008	0.007	0.280
DBP (mmHg)	0.003	0.003	0.365	0.005	0.002	0.028	0.001	0.011	0.939
TG (mmol/L)	−0.008	0.020	0.700	−0.029	0.017	0.084	0.001	0.064	0.984
TC (mmol/L)	0.078	0.026	0.002	0.051	0.017	0.003	−0.041	0.090	0.648
LDL-C (mmol/L)	0.147	0.035	<0.001	0.075	0.024	0.002	0.410	0.121	0.001
HDL-C (mmol/L)	−0.401	0.076	<0.001	−0.134	0.050	0.007	−0.780	0.271	0.004
ALT (U/L)	−0.002	0.001	0.244	−0.004	0.001	0.332	−0.012	0.007	0.085
AST (U/L)	0.002	0.002	0.486	0.001	0.002	0.553	−0.005	0.014	0.745
ALP (U/L)	0.002	0.001	0.072	0.001	0.001	0.314	−0.004	0.005	0.444
GGT (U/L)	0.001	0.001	0.105	0.000	0.001	0.314	0.002	0.002	0.531
BUN (mmol/L)	0.008	0.005	0.099	0.005	0.005	0.322	0.003	0.018	0.855
Scr (μmol/L)	0.000	0.001	0.940	0.003	0.001	0.002	0.002	0.005	0.619
SUA (μmol/L)	0.001	0.000	0.057	−0.000	0.000	0.585	0.000	0.001	0.732

*BMI, body mass index; FBG, fasting blood glucose; SBP, systolic blood pressure; DBP, diastolic blood pressure; HCT, hematocrit; MCV, mean corpusular volume; TG, triglyceride; TC, serum total cholesterol; LDL-C, low-density lipoprotein cholesterol; HDL-C, high-density lipoprotein cholesterol; ALT, alanine aminotransferase; AST, aspartate aminotransferase; ALP, alkaline phosphatase; GGT, gama-glutamyl transpeptadase; BUN, blood urea nitrogen SUA, serum uric acid; Scr, serum creatinine.*

**FIGURE 2:**
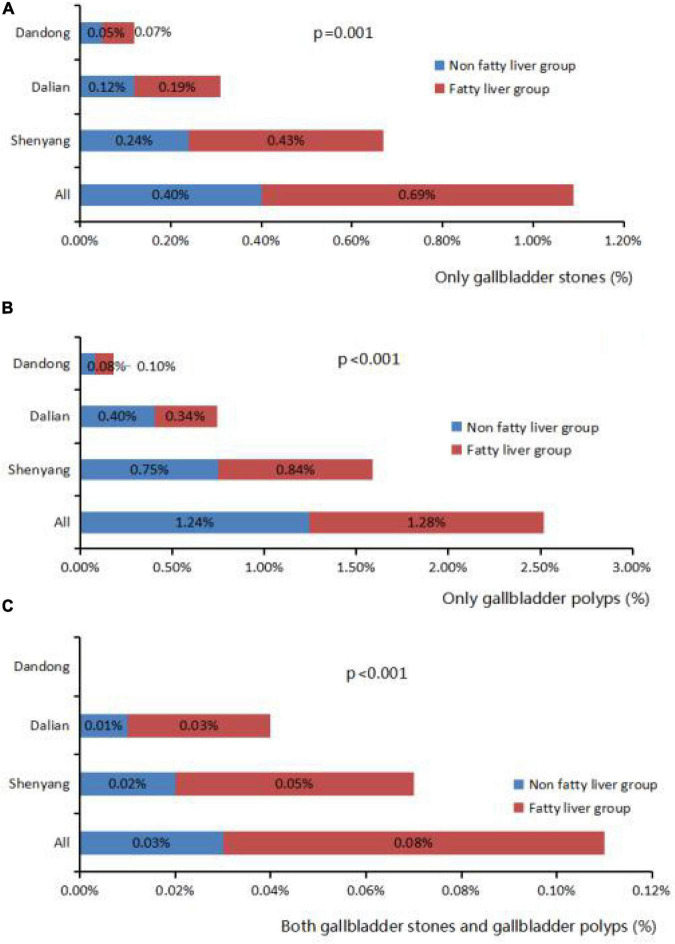
Correlation fatty liver and gallbladder stone and gallbladder polyps. **(A)** The proportion of gallbladder stones in fatty liver group and non-fatty liver group in different cities. **(B)** The proportion of gallbladder polyps in fatty liver group and non-fatty liver group in different cities. **(C)** The proportion of both gallbladder stones and polyps in fatty liver group and non-fatty liver group in different cities.

Overweight, obesity, hypertension, diabetes, fatty liver, hypertriglyceridemia, and hypercholesterolemia prevalence were higher in patients with gallbladder stones than those without (*P* < 0.001). Similar results were observed for patients with gallbladder polyps, except that there were no differences in obesity, hypertriglyceridemia, or diabetes (Table 4). 91,837 Patients have fatty liver by ultrasound, accounting for 33.10%. Patients with fatty liver had a higher prevalence of gallbladder polyps and gallbladder stones than those without fatty liver, and this was seen in all cities (*P* < 0.001) (Figure 2).

**TABLE 4:** Prevalence of metabolic associated disease in gallbladder stones and gallbladder polyps.

	sGS	sGP	GSP	nGSP	*P*^1^ value	*P*^2^ value	*P*^3^ value
Overweight(n/%)	852/61.6%	1762/50.8%	87/71.3%	23751/45.8%	<0.001	<0.001	<0.001
Obesity(n/%)	318/23%	445/12.8%	33/27%	7226/13.9%	<0.001	0.071	<0.001
Hypertension(n/%)	390/28.18%	557/16.05%	36/29.51%	7491/14.43%	<0.001	0.009	<0.001
Diabetes(n/%)	123/8.89%	125/3.60%	12/9.87%	1745/3.36%	<0.001	0.447	<0.001
Fatty liver(n/%)	637/46.03%	1172/33.78%	70/57.38%	16393/31.58%	<0.001	0.007	<0.001
Hypertriglyceridemia(n/%)	424/30.64%	835/24.03%	47/38.52%	11807/22.74%	<0.001	0.073	<0.001
Hypercholesterolemia(n/%)	159/11.49%	283/8.16%	8/6.56%	3734/7.19%	<0.001	0.034	0.876

*P^1^ value, comparision between sGS group and nGSP group; P^2^ value, comparision between sGP group and nGSP group; P^3^ value, comparision between GSP group and nGSP group.*

## Discussion

The incidence of gallbladder stones differs by country likely because of differences in geographical environment and lifestyle. The incidence in European and American countries is about 5–8% and slightly higher in China (6). The prevalence also differed between regions of China with Xinjiang having the highest prevalence of about 15%. The prevalence of gallbladder polyps in adults ranges from 0.3 to –12.3% (7). Other studies have reported extreme variations in the prevalence of gallbladder polyps by nationality. The prevalence in Japan, Germany, India, and Korea was 5.6, 1.5, 0.32, and 2.94%, respectively. Gallbladder polyps were reported in 6.7% of Chinese participants, but recent studies have shown a higher prevalence (8). A survey showed that the prevalence of gallbladder stones and polyps in Liaoning was 2.30 and 6.31%, respectively. These incidences were significantly lower than those seen in other parts of China. But whether Liaoning Province has lower incidences remains to be verified. Findings from the current study showed an increasing yearly prevalence of gallbladder stones and polyps. This is likely because of higher detection rates that have resulted from increasing health awareness and a higher number of medical examinations. Changes in diet have also led to an increase in exposure to fatty elements that have affected cholesterol metabolism and promoted disease.

In this study, the prevalence of gallbladder stones was higher among females than males. Hormones such as estrogen and progesterone play an important role in high-risk diseases that are specific to women. Estrogen causes cholesterol supersaturation by increasing cholesterol secretion while progesterone inhibits gallbladder contraction and causes cholestasis (9, 10).

Metabolic syndrome is closely related to gallbladder disease, including gallstones (11). Metabolic syndrome includes metabolic disorders of proteins, fats, carbohydrates, and other substances, and is characterized by hypertension, hyperlipidemia, abdominal obesity, and insulin resistance (12). Logistic regression analysis found that fasting blood glucose is a risk factor for gallstones. A rise in fasting glucose occurs because of insulin resistance or a relative decrease in insulin secretion. Insulin resistance increases the secretion of cholesterol, reduces the synthesis of bile acid, and lowers the cholecystokinin response, slowing gallbladder movement and causing cholesterol to remain in the gallbladder, thereby increasing the risk of stones and polyp formation (13, 14). Increased blood sugar levels also inhibit gallbladder contraction and bile secretion, promoting the formation of stones (1).

In the current study, high BMI (overweight) increased the risk of gallbladder stones and polyps, which is consistent with findings from other studies (15, 16). There are several possible mechanisms by which high BMI increases the risk of gallbladder stones and polyps: (1) high BMI is associated with a larger gallbladder and increased cholesterol synthase activity that promotes bile cholesterol supersaturation and the formation of gallstones and polyps (17), (2) high BMI causes slower metabolism of lipids and endogenous hormones, reducing gallbladder movement and increase risk of gallbladder stones and polyps (18), and (3) high BMI can lead to insulin insensitivity and resistance, additional risk factors for gallstone formation (19).

Hypertension is one of the diagnostic criteria for metabolic syndrome. Results from this study showed that high systolic blood pressure was associated with an increased prevalence of gallbladder stones. While no clear mechanism has been defined, this association may be explained by the increase in insulin resistance observed in people with hypertension (20). However, the correlation between blood pressure and gallbladder polyps remains unclear. In a study conducted at Capital Medical University, an increase in diastolic blood pressure increased the prevalence of gallbladder polyps (21), another study found no link between them (22). Studies also show different results for the metabolic indicators of total cholesterol, triglycerides, LDL-C, and HDL-C (11, 23). Some have shown that triglycerides reduce the gallbladder’s contractile function, resulting in cholesterol accumulation and the formation of crystals (24). Elevated cholesterol has been shown to induce the expression of inflammatory factors, which may cause cholecystitis and induce gallstone formation (23). In the current study, logistic regression showed that only LDL-C and HDL-C were associated with gallbladder disease, with HDL-C serving as a protective factor. While low HDL-C causes insulin resistance, the high catabolism rate of HDL-C increases the rate of bile cholesterol secretion (24).

Fatty liver is the most prevalent liver disease in China. The risk factors of fatty liver are similar to those of gallbladder disease, including alcohol use, obesity, diabetes, and metabolic syndrome (25). The current study compared the prevalence of gallbladder stones and polyps in fatty liver and non-fatty liver groups in three cities of Liaoning Province to assess the relationship between fatty liver and gallbladder disease in this region. The prevalence of gallbladder stones and polyps was significantly higher in the fatty liver group than in the non-fatty liver group suggesting that fatty liver increases the prevalence of gallbladder stones and polyps. Similarly, gallbladder stones and polyps are risk factors of fatty liver diseases. Shen et al. conducted a meta-analysis of 12 studies in 5 major databases (26), and found that the incidence of gallstones in the non-alcoholic fatty liver group was 17% and the OR value was 1.4 (95% CI, 1.23–1.59) in the correlation analysis. Non-alcoholic fatty liver and cholelithiasis were significantly related. Liu et al. conducted a cohort study on the urban population of China (27). Results indicated that the prevalence of cholelithiasis in 4,713 patients with fatty liver and 6,487 patients with non-fatty liver were 6.1 and 3.2%, respectively. The RR value was 1.71 (95% CI, 1.245–2.341) in a related study. Lim et al. also showed a significant relationship between gallbladder polyps and fatty liver. But there are also reports with different findings (28). A meta-analysis of the risk factors for gallbladder polyps conducted by Yamin et al. indicated that the incidence was not related to the occurrence of fatty liver (29). Thus, this relationship requires further exploration. Possible mechanisms for the association between fatty liver and gallbladder polyps and stones include (1) poor normal lipid metabolism of fatty liver, and reduced ability of the liver to synthesize phospholipids, resulting in a relatively high ratio of cholesterol to phospholipids that leads to a large amount of cholesterol precipitated on the wall, leading to gallbladder stones or polyps (30), (2) poor synthetic secretion function of liver cells, resulting in a decrease in the secretion of bile acids and other substances that promote gallbladder movement, slowing the bile emptying rate and promoting the formation of stones and polyps, and (3) increased formation of fat due to poor lipid metabolism, resulting in insulin resistance (31).

In summary, the prevalence of gallbladder stones and polyps in Liaoning is related to sex, economic status of the city of residence, BMI, and metabolic indicators. Longitudinal studies will be required to determine the factors associated with disease development.

## Data Availability Statement

The original contributions presented in the study are included in the article/supplementary material, further inquiries can be directed to the corresponding author/s.

## Ethics Statement

The studies involving human participants were reviewed and approved by the Ethics Committee of the First Hospital of China Medical University ([2020]2020-257). The patients/participants provided their written informed consent to participate in this study.

## Author Contributions

XZ: writing-original draft preparation. YL: writing-review and editing. LG and HT: data collecion. All authors contributed to the article and approved the submitted version.

## Conflict of Interest

The authors declare that the research was conducted in the absence of any commercial or financial relationships that could be construed as a potential conflict of interest.

## Publisher’s Note

All claims expressed in this article are solely those of the authors and do not necessarily represent those of their affiliated organizations, or those of the publisher, the editors and the reviewers. Any product that may be evaluated in this article, or claim that may be made by its manufacturer, is not guaranteed or endorsed by the publisher.

## References

[1] Di Ciaula A Garruti G Frühbeck G De Angelis M de Bari O Wang DQ The role of diet in the pathogenesis of cholesterol gallstones. *Curr Med Chem.* (2019) 26:3620–38. 10.2174/0929867324666170530080636 28554328 PMC8118138

[2] Kim YK Kwon OS Her KH. The grade of nonalcoholic fatty liver disease is an independent risk factor for gallstone disease: an observational study. *Medicine.* (2019) 98:e16018. 10.1097/MD.0000000000016018 31277096 PMC6635222

[3] Sun N Zuo W Zhou Y Cheng Y Cheng S Zhou J Acupuncture for biliary colic: a systematic review protocol. *BMJ Open.* (2021) 11:e041931. 10.1136/bmjopen-2020-041931 33455934 PMC7813305

[4] Vila M Lladó L Ramos E. Management and treatment of gallbladder polyps. *Med Clin.* (2018) 150:487–91. 10.1016/j.medcli.2017.12.003 29426789

[5] Valibouze C El Amrani M Truant S Leroy C Millet G Pruvot FR The management of gallbladder polyps. *J Visc Surg.* (2020) 157:410–7. 10.1016/j.jviscsurg.2020.04.008 32473822

[6] van Dijk AH de Reuver PR Besselink MG van Laarhoven KJ Harrison EM Wigmore SJ Assessment of available evidence in the management of gallbladder and bile duct stones: a systematic review of international guidelines. *HPB.* (2017) 19:297–309. 10.1016/j.hpb.2016.12.011 28117228

[7] McCain RS Diamond A Jones C Coleman HG. Current practices and future prospects for the management of gallbladder polyps: a topical review. *World J Gastroenterol.* (2018) 24:2844–52. 10.3748/wjg.v24.i26.2844 30018479 PMC6048427

[8] Ali TA Abougazia AS Alnuaimi AS Mohammed MAM. Prevalence and risk factors of gallbladder polyps in primary health care centers among patients examined by abdominal ultrasonography in Qatar: a case-control study. *Qatar Med J.* (2021) 2021:48. 10.5339/qmj.2021.48 34660216 PMC8501236

[9] Weerakoon HT Ranasinghe JG Navaratna A Sivakanesan R Galketiya KB Rosairo S. Can the type of gallstones be predicted with known possible risk factors?: a comparison between mixed cholesterol and black pigment stones. *BMC Gastroenterol.* (2014) 14:88. 10.1186/1471-230X-14-88 24884475 PMC4017087

[10] Yang JL Huang JJ Cheng N Zhang S Liu SM Huang WY Sex-specific and dose-response relationship between the incidence of gallstones and components of the metabolic syndrome in jinchang cohort: a prospective study. *Biomed Environ Sci.* (2020) 33:633–8. 10.3967/bes2020.084 32933617

[11] Almobarak AO Jervase A Fadl AA Garelnabi NIA Hakem SA Hussein TM The prevalence of diabetes and metabolic syndrome and associated risk factors in Sudanese individuals with gallstones: a cross sectional survey. *Transl Gastroenterol Hepatol.* (2020) 5:14. 10.21037/tgh.2019.10.09 32258518 PMC7063511

[12] Saklayen MG. The global epidemic of the metabolic syndrome. *Curr Hypertens Rep.* (2018) 20:12. 10.1007/s11906-018-0812-z 29480368 PMC5866840

[13] Cortés VA Barrera F Nervi F. Pathophysiological connections between gallstone disease, insulin resistance, and obesity. *Obes Rev.* (2020) 21:e12983. 10.1111/obr.12983 31814283

[14] Di Ciaula A Wang DQ Portincasa P. An update on the pathogenesis of cholesterol gallstone disease. *Curr Opin Gastroenterol.* (2018) 34:71–80. 10.1097/MOG.0000000000000423 29283909 PMC8118137

[15] Lee JK Hahn SJ Kang HW Jung JG Choi HS Lee JH Visceral obesity is associated with gallbladder polyps. *Gut Liver.* (2016) 10:133–9. 10.5009/gnl14506 26260756 PMC4694745

[16] Lim J Wirth J Wu K Giovannucci E Kraft P Turman C Obesity, adiposity, and risk of symptomatic gallstone disease according to genetic susceptibility. *Clin Gastroenterol Hepatol.* (2021) 2021:44. 10.1016/j.cgh.2021.06.044 34217876 PMC8720320

[17] Liu T Wang W Ji Y Wang Y Liu X Cao L Association between different combination of measures for obesity and new-onset gallstone disease. *PLoS One.* (2018) 13:e0196457. 10.1371/journal.pone.0196457 29772027 PMC5957348

[18] Nam SY. Obesity-related digestive diseases and their pathophysiology. *Gut Liver.* (2017) 11:323–34. 10.5009/gnl15557 27890867 PMC5417774

[19] Yuan S Gill D Giovannucci EL Larsson SC. Obesity, type 2 diabetes, lifestyle factors, and risk of gallstone disease: a mendelian randomization investigation. *Clin Gastroenterol Hepatol.* (2022) 20:e529–37. 10.1016/j.cgh.2020.12.034 33418132

[20] da Silva AA do Carmo JM Li X Wang Z Mouton AJ Hall JE. Role of hyperinsulinemia and insulin resistance in hypertension: metabolic syndrome revisited. *Can J Cardiol.* (2020) 36:671–82. 10.1016/j.cjca.2020.02.066 32389340 PMC7219403

[21] Xu Q Tao LY Wu Q Gao F Zhang FL Yuan L Prevalences of and risk factors for biliary stones and gallbladder polyps in a large Chinese population. *HPB.* (2012) 14:373–81. 10.1111/j.1477-2574.2012.00457.x 22568413 PMC3384861

[22] Mao YS Mai YF Li FJ Zhang YM Hu KM Hong ZL Prevalence and risk factors of gallbladder polypoid lesions in Chinese petrochemical employees. *World J Gastroenterol.* (2013) 19:4393–9. 10.3748/wjg.v19.i27.4393 23885152 PMC3718909

[23] Gu Q Zhou G Xu T. Risk factors for gallstone disease in Shanghai. *Medicine.* (2020) 99:e18754. 10.1097/md.0000000000018754 32011459 PMC7220401

[24] Smelt AH. Triglycerides and gallstone formation. *Clin Chim Acta.* (2010) 411:1625–31. 10.1016/j.cca.2010.08.003 20699090

[25] Kichloo A Solanki S Haq KF Dahiya D Bailey B Solanki D Association of non-alcoholic fatty liver disease with gallstone disease in the United States hospitalized patient population. *World J Gastrointest Pathophysiol.* (2021) 12:14–24. 10.4291/wjgp.v12.i2.14 33815863 PMC8008957

[26] Shen SS Gong JJ Wang XW Chen L Qin S Huang LF Promotional effect of nonalcoholic fatty liver disease on Gallstone disease: a systematic review and meta-analysis. *Turk J Gastroenterol.* (2017) 28:31–9. 10.5152/tjg.2016.0357 27991855

[27] Liu J Lin H Zhang C Wang L Wu S Zhang D Non-alcoholic fatty liver disease associated with gallstones in females rather than males: a longitudinal cohort study in Chinese urban population. *BMC Gastroenterol.* (2014) 14:213. 10.1186/s12876-014-0213-y 25496394 PMC4273434

[28] Lim SH Kim D Kang JH Song JH Yang SY Yim JY Hepatic fat, not visceral fat, is associated with gallbladder polyps: a study of 2643 healthy subjects. *J Gastroenterol Hepatol.* (2015) 30:767–74. 10.1111/jgh.12841 25376159

[29] Yamin Z Xuesong B Guibin Y Liwei L Fei L. Risk factors of gallbladder polyps formation in East Asian population: a meta-analysis and systematic review. *Asian J Surg.* (2020) 43:52–9. 10.1016/j.asjsur.2019.03.015 31109764

[30] Ahn DW Jeong JB Kang J Kim SH Kim JW Kim BG Fatty liver is an independent risk factor for gallbladder polyps. *World J Gastroenterol.* (2020) 26:6979–92. 10.3748/wjg.v26.i44.6979 33311944 PMC7701938

[31] Chang Y Noh YH Suh BS Kim Y Sung E Jung HS Bidirectional association between nonalcoholic fatty liver disease and gallstone disease: a cohort study. *J Clin Med.* (2018) 7:458. 10.3390/jcm7110458 30469392 PMC6262563

